# (*E*)-2-(4-Chloro­phen­oxy)-*N*′-(pyridin-4-yl­methyl­idene)acetohydrazide

**DOI:** 10.1107/S1600536812045989

**Published:** 2012-12-08

**Authors:** Xiao-jin Rao, Wen-Shi Wu, Chuan-hui Li, Yu-min Huang

**Affiliations:** a College of Materials Science and Engineering, Huaqiao University, Xiamen, Fujian 361021, People’s Republic of China

## Abstract

In the title compound, C_14_H_12_ClN_3_O_2_, the acyl­hydrazone base [C(=O)—N—N=C] is essentially planar, with an r.m.s. deviation of 0.0095 Å, and makes a dihedral angle of 12.52 (10)°with the pyridine ring. In the crystal, molecules are linked *via* pairs of N—H⋯O hydrogen bonds, forming inversion dimers with an *R*_2_^2^(8) graph-set motif. The dimers are linked *via* C—H⋯π interactions forming chains along [101].

## Related literature

For chemical properties of hydrazides, see: Narayana *et al.* (2005 ▶); Liu *et al.* (2006 ▶). For the synthesis and structure of eth­yl(4-chloro­phen­oxy)acetate, see: Dutkiewicz *et al.* (2009 ▶). For graph-set motifs, see: Etter *et al.* (1990 ▶) and for classification of hydrogen bonds, see: Gilli & Gilli (2009 ▶). 
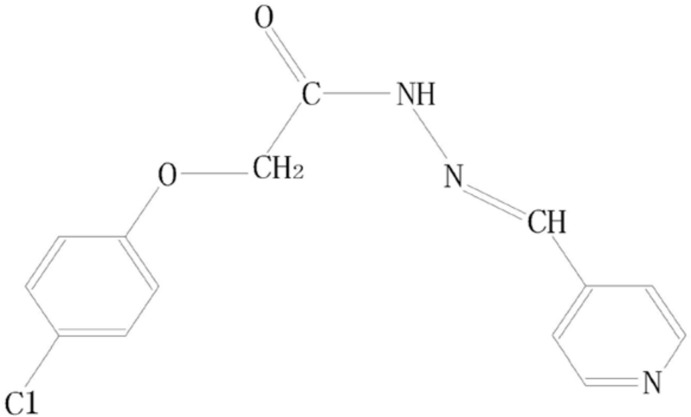


## Experimental

### 

#### Crystal data

C_14_H_12_ClN_3_O_2_
*M* *_r_* = 289.72
Monoclinic, 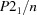

*a* = 13.059 (4) Å
*b* = 5.3567 (16) Å
*c* = 19.175 (6) Å
β = 104.586 (5)°
*V* = 1298.2 (7) Å^3^
*Z* = 4
Mo *K*α radiation
μ = 0.30 mm^−1^
*T* = 173 K
0.53 × 0.21 × 0.14 mm

#### Data collection

Bruker SMART APEX diffractometer
Absorption correction: multi-scan (*SADABS*; Sheldrick, 1996 ▶) *T*_min_ = 0.927, *T*_max_ = 0.959
7114 measured reflections
2796 independent reflections
2437 reflections with *I* > 2σ(*I*)
*R*_int_ = 0.033

#### Refinement

*R*[*F* ^2^ > 2σ(*F* ^2^)] = 0.041
*wR*(*F* ^2^) = 0.104
*S* = 1.05
2796 reflections
184 parameters
H atoms treated by a mixture of independent and constrained refinement
Δρ_max_ = 0.27 e Å^−3^
Δρ_min_ = −0.24 e Å^−3^

### 

Data collection: *SMART* (Bruker, 1999 ▶); cell refinement: *SAINT* (Bruker, 1999 ▶); data reduction: *SAINT*; program(s) used to solve structure: *SHELXS97* (Sheldrick, 2008 ▶); program(s) used to refine structure: *SHELXL97* (Sheldrick, 2008 ▶); molecular graphics: *SHELXTL* (Sheldrick, 2008 ▶); software used to prepare material for publication: *SHELXTL*.

## Supplementary Material

Crystal structure: contains datablock(s) I, global. DOI: 10.1107/S1600536812045989/fb2275sup1.cif

Structure factors: contains datablock(s) I. DOI: 10.1107/S1600536812045989/fb2275Isup2.hkl

Supplementary material file. DOI: 10.1107/S1600536812045989/fb2275Isup3.cml

Additional supplementary materials:  crystallographic information; 3D view; checkCIF report

## Figures and Tables

**Table 1:** Hydrogen-bond geometry (Å, °) *Cg*2 is the centroid of the C3–C8 phenyl ring.

*D*—H⋯*A*	*D*—H	H⋯*A*	*D*⋯*A*	*D*—H⋯*A*
N1—H1*A*⋯O1^i^	0.885 (19)	1.96 (2)	2.8423 (19)	172.9 (18)
C2—H2*A*⋯*Cg*2^ii^	0.97	2.88	3.676 (2)	140

Symmetry codes: (i) 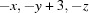; (ii) 
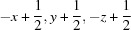
.

**Fig. 1.**
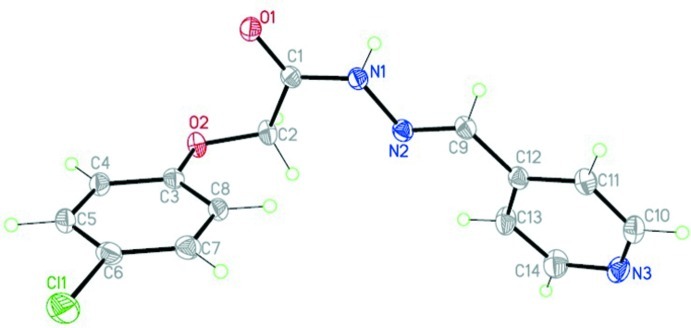
The title molecule with the displacement ellipsoids at the 40% probability level.

**Fig. 2.**
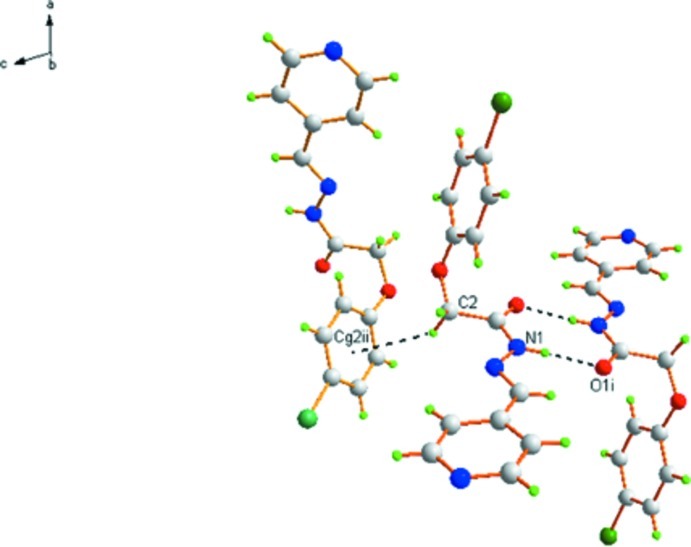
The hydrogen bonds in the title compound. Hydrogen bonds are shown as dashed lines. O1A is a symmetry equivalent to O1 transformed by x, -y + 3, -z. Cg2ii is a centroid of the phenyl ring C3\C4\C5\C6\C7\C8; symmetry code (ii): -x + 1/2, y + 1/2, -z + 1/2.

## supplementary crystallographic information

### Comment

Hydrazides usually serve as precursors in the synthesis of several heterocyclic systems (Narayana *et al.*, 2005). Some substituted hydrazides are used as intermediates in many pharmaceutically important compounds (Liu *et al.*, 2006). A new hydrazide, (*E*)-2-(4-chlorophenoxy)-*N*'-(pyridin-4-ylmethylene)acetohydrazide, *C*_14_H_12_Cl_1_O_2_N_3_, has been synthesized and its crystal structure is reported here (Fig. 1).

In the title molecule, the principal cohesion interactions are N—H···O hydrogen bonds of moderate strength (Gilli & Gilli, 2009) which link the molecules into a dimer with the graph-set motif (Etter *et al.*, 1990) *R*^2^_2_(8) (Tab. 1). Moreover, there are present C—H···π-electron ring interactions in the structure (Fig. 2). The acylhydrazone base is essentially planar with the r.m.s. deviation from planarity which equals to 0.0095 Å. The pyridine ring and the acylhydrazone base [C1(═O1)—N1—N2═C9] contain the angle 12.52 (10)°.

### Experimental

The synthesis of the title structure proceeded in three steps.

First, concentrated H_2_SO_4_ (98 weight %, 1.4 ml) was added slowly while stirring to a mixture of 4-chlorophenoxyacetic acid (11.2 g, 0.06 mol) in ethanol (99.7 volume %, 120 ml). The mixture was left to reflux for 6 h at 359 K. Then 34.2 ml of 98.5 weight % of tris(2-hydroxyethyl)amine (trolamine) were added dropwise into the mixture while stirring in order to neutralize the mixture. Then the ethanol in the mixture was removed by reduced pressure distillation (335 K, about 0.003 MPa). What has left was poured into 100 ml of water heated to 321 K. The white precipitate (12.21 g) of ethyl(4-chlorophenoxy)acetate was filtered and washed.

Second step consisted in the synthesis of 2-(4-chlorophenoxy)acetohydrazide. It was carried out according to the method applied by Dutkiewicz *et al.* (2009). A mixture of ethyl(4-chlorophenoxy)acetate (8.56 g, 0.04 mol) and 50 ml of hydrazine hydrate (85 weight %) was refluxed over water bath for 5 h at 365 K. The precipitate was filtered off and recrystallized from ethanol (99.7 volume %) yielding plate-like colourless crystals of 2-(4-chlorophenoxy)acetohydrazide.

Finally, the title compound was synthesized by adding 4-pyridinecarboxaldehyde (5 ml) slowly to a mixture of 2-(4-chlorophenoxy) acetohydrazide in ethanol (99.7 volume %, 20 ml) and water (15 ml) while stirring. Then the mixture was refluxed for 3.5 h at room temperature. Prismatic colourless crystals with the size about that of the used sample formed in 24 h.

### Refinement

All the hydrogen atoms were identified in the difference electron density map, nevertheless the aryl and methylene H atoms were situated into idealized positions and constrained to ride on their parent atoms with C—H = 0.93 and 0.97 Å for aryl and methylene H atoms, respectively, with *U*_iso_(H_aryl_/_methylene_) = 1.2*U*_eq_(C_aryl_/_methylene_). The positional parameters of the secondary amine H atom were refined freely while its isotropic displacement parameter was constrained as 1.2 multiple of the equivalent isotropic parameter of its carrier atom.

### Crystal data

**Table:**

C_14_H_12_ClN_3_O_2_	*F*(000) = 600
*M**_r_* = 289.72	*D*_x_ = 1.482 Mg m^−^^3^
Monoclinic, *P*2_1_/*n*	Mo *K*α radiation, λ = 0.71073 Å
Hall symbol: -P 2yn	Cell parameters from 3317 reflections
*a* = 13.059 (4) Å	θ = 2.2–27.9°
*b* = 5.3567 (16) Å	µ = 0.30 mm^−^^1^
*c* = 19.175 (6) Å	*T* = 173 K
β = 104.586 (5)°	Prism, colourless
*V* = 1298.2 (7) Å^3^	0.53 × 0.21 × 0.14 mm
*Z* = 4	

### Data collection

**Table:**

Bruker SMART APEX diffractometer	2796 independent reflections
Radiation source: fine-focus sealed tube	2437 reflections with *I* > 2σ(*I*)
Graphite monochromator	*R*_int_ = 0.033
ω scans	θ_max_ = 27.0°, θ_min_ = 1.7°
Absorption correction: multi-scan (*SADABS*; Sheldrick, 1996)	*h* = −16→16
*T*_min_ = 0.927, *T*_max_ = 0.959	*k* = −6→6
7114 measured reflections	*l* = −24→15

### Refinement

**Table:**

Refinement on *F*^2^	Primary atom site location: structure-invariant direct methods
Least-squares matrix: full	Secondary atom site location: difference Fourier map
*R*[*F*^2^ > 2σ(*F*^2^)] = 0.041	Hydrogen site location: difference Fourier map
*wR*(*F*^2^) = 0.104	H atoms treated by a mixture of independent and constrained refinement
*S* = 1.05	*w* = 1/[σ^2^(*F*_o_^2^) + (0.037*P*)^2^ + 0.573*P*] where *P* = (*F*_o_^2^ + 2*F*_c_^2^)/3
2796 reflections	(Δ/σ)_max_ < 0.001
184 parameters	Δρ_max_ = 0.27 e Å^−^^3^
0 restraints	Δρ_min_ = −0.24 e Å^−^^3^
45 constraints	

### Special details

**Table:**

Geometry. All e.s.d.'s (except the e.s.d. in the dihedral angle between two l.s. planes) are estimated using the full covariance matrix. The cell e.s.d.'s are taken into account individually in the estimation of e.s.d.'s in distances, angles and torsion angles; correlations between e.s.d.'s in cell parameters are only used when they are defined by crystal symmetry. An approximate (isotropic) treatment of cell e.s.d.'s is used for estimating e.s.d.'s involving l.s. planes.
Refinement. Refinement of *F*^2^ against ALL reflections. The weighted *R*-factor *wR* and goodness of fit *S* are based on *F*^2^, conventional *R*-factors *R* are based on *F*, with *F* set to zero for negative *F*^2^. The threshold expression of *F*^2^ > σ(*F*^2^) is used only for calculating *R*-factors(gt) *etc*. and is not relevant to the choice of reflections for refinement. *R*-factors based on *F*^2^ are statistically about twice as large as those based on *F*, and *R*- factors based on ALL data will be even larger.

### Fractional atomic coordinates and isotropic or equivalent isotropic displacement parameters (Å^2^)

**Table:**

	*x*	*y*	*z*	*U*_iso_*/*U*_eq_	
C1	0.07691 (12)	1.4108 (3)	0.10391 (8)	0.0216 (3)	
C2	0.12993 (12)	1.3618 (3)	0.18254 (8)	0.0244 (3)	
H2A	0.0959	1.4606	0.2127	0.029*	
H2B	0.1224	1.1870	0.1934	0.029*	
C3	0.29987 (12)	1.2712 (3)	0.16797 (8)	0.0210 (3)	
C4	0.40322 (12)	1.3495 (3)	0.17631 (8)	0.0242 (3)	
H4A	0.4260	1.4979	0.2004	0.029*	
C5	0.47256 (13)	1.2089 (3)	0.14905 (9)	0.0269 (4)	
H5A	0.5420	1.2616	0.1545	0.032*	
C6	0.43774 (13)	0.9893 (3)	0.11357 (9)	0.0267 (4)	
C7	0.33550 (13)	0.9090 (3)	0.10505 (8)	0.0262 (4)	
H7A	0.3130	0.7607	0.0808	0.031*	
C8	0.26625 (12)	1.0497 (3)	0.13270 (8)	0.0230 (3)	
H8A	0.1971	0.9953	0.1276	0.028*	
C9	−0.12003 (12)	0.9586 (3)	0.06945 (8)	0.0236 (3)	
H9A	−0.1455	0.9987	0.0210	0.028*	
C10	−0.28617 (13)	0.4072 (3)	0.08435 (9)	0.0287 (4)	
H10A	−0.3333	0.3023	0.0532	0.034*	
C11	−0.24060 (12)	0.5978 (3)	0.05463 (9)	0.0262 (4)	
H11A	−0.2573	0.6204	0.0050	0.031*	
C12	−0.16955 (12)	0.7556 (3)	0.09964 (8)	0.0231 (3)	
C13	−0.14931 (14)	0.7123 (4)	0.17301 (9)	0.0326 (4)	
H13A	−0.1027	0.8143	0.2054	0.039*	
C14	−0.19892 (15)	0.5172 (4)	0.19725 (10)	0.0368 (4)	
H14A	−0.1840	0.4903	0.2467	0.044*	
Cl1	0.52572 (4)	0.80977 (9)	0.08079 (3)	0.04032 (16)	
N1	−0.00592 (10)	1.2696 (3)	0.07166 (7)	0.0233 (3)	
H1A	−0.0369 (15)	1.304 (4)	0.0261 (10)	0.028*	
N2	−0.04318 (10)	1.0820 (3)	0.10753 (7)	0.0232 (3)	
N3	−0.26732 (11)	0.3633 (3)	0.15439 (8)	0.0308 (3)	
O1	0.10839 (9)	1.5774 (2)	0.07075 (6)	0.0277 (3)	
O2	0.23832 (8)	1.4241 (2)	0.19755 (6)	0.0237 (3)	

### Atomic displacement parameters (Å^2^)

**Table:**

	*U*^11^	*U*^22^	*U*^33^	*U*^12^	*U*^13^	*U*^23^
C1	0.0174 (7)	0.0246 (9)	0.0239 (7)	0.0029 (6)	0.0071 (6)	−0.0003 (6)
C2	0.0182 (7)	0.0337 (9)	0.0218 (7)	−0.0036 (7)	0.0060 (6)	−0.0021 (7)
C3	0.0208 (8)	0.0261 (8)	0.0147 (7)	−0.0005 (6)	0.0022 (6)	0.0038 (6)
C4	0.0219 (8)	0.0257 (9)	0.0233 (7)	−0.0037 (6)	0.0028 (6)	0.0009 (6)
C5	0.0187 (8)	0.0312 (9)	0.0307 (8)	−0.0020 (7)	0.0063 (6)	0.0073 (7)
C6	0.0253 (8)	0.0289 (9)	0.0274 (8)	0.0077 (7)	0.0095 (6)	0.0069 (7)
C7	0.0281 (8)	0.0244 (9)	0.0237 (8)	0.0006 (7)	0.0023 (6)	0.0009 (6)
C8	0.0187 (7)	0.0258 (9)	0.0224 (7)	−0.0021 (6)	0.0015 (6)	0.0040 (6)
C9	0.0200 (7)	0.0278 (9)	0.0228 (7)	0.0012 (6)	0.0048 (6)	0.0006 (6)
C10	0.0232 (8)	0.0305 (10)	0.0335 (9)	−0.0046 (7)	0.0093 (7)	−0.0074 (7)
C11	0.0225 (8)	0.0317 (10)	0.0251 (8)	−0.0006 (7)	0.0076 (6)	−0.0033 (7)
C12	0.0175 (7)	0.0260 (9)	0.0266 (8)	0.0004 (6)	0.0072 (6)	−0.0007 (6)
C13	0.0308 (9)	0.0391 (11)	0.0260 (8)	−0.0120 (8)	0.0036 (7)	−0.0024 (7)
C14	0.0384 (10)	0.0454 (12)	0.0269 (9)	−0.0093 (9)	0.0087 (7)	0.0041 (8)
Cl1	0.0365 (3)	0.0378 (3)	0.0524 (3)	0.0111 (2)	0.0219 (2)	0.0032 (2)
N1	0.0197 (7)	0.0277 (8)	0.0214 (7)	−0.0029 (6)	0.0032 (5)	0.0041 (6)
N2	0.0184 (6)	0.0262 (7)	0.0261 (7)	−0.0011 (5)	0.0074 (5)	0.0019 (5)
N3	0.0269 (7)	0.0316 (8)	0.0358 (8)	−0.0040 (6)	0.0115 (6)	0.0008 (6)
O1	0.0238 (6)	0.0315 (7)	0.0260 (6)	−0.0056 (5)	0.0029 (5)	0.0043 (5)
O2	0.0176 (5)	0.0304 (6)	0.0227 (5)	−0.0028 (5)	0.0042 (4)	−0.0048 (5)

### Geometric parameters (Å, º)

**Table:**

C1—O1	1.2251 (19)	C8—H8A	0.9300
C1—N1	1.337 (2)	C9—N2	1.268 (2)
C1—C2	1.515 (2)	C9—C12	1.458 (2)
C2—O2	1.4117 (18)	C9—H9A	0.9300
C2—H2A	0.9700	C10—N3	1.324 (2)
C2—H2B	0.9700	C10—C11	1.376 (2)
C3—O2	1.3667 (19)	C10—H10A	0.9300
C3—C8	1.382 (2)	C11—C12	1.385 (2)
C3—C4	1.384 (2)	C11—H11A	0.9300
C4—C5	1.378 (2)	C12—C13	1.384 (2)
C4—H4A	0.9300	C13—C14	1.371 (3)
C5—C6	1.378 (3)	C13—H13A	0.9300
C5—H5A	0.9300	C14—N3	1.335 (2)
C6—C7	1.373 (2)	C14—H14A	0.9300
C6—Cl1	1.7331 (17)	N1—N2	1.3740 (19)
C7—C8	1.381 (2)	N1—H1A	0.885 (19)
C7—H7A	0.9300		
			
O1—C1—N1	120.66 (14)	C3—C8—H8A	120.1
O1—C1—C2	120.91 (14)	N2—C9—C12	121.74 (14)
N1—C1—C2	118.43 (14)	N2—C9—H9A	119.1
O2—C2—C1	110.20 (12)	C12—C9—H9A	119.1
O2—C2—H2A	109.6	N3—C10—C11	124.23 (16)
C1—C2—H2A	109.6	N3—C10—H10A	117.9
O2—C2—H2B	109.6	C11—C10—H10A	117.9
C1—C2—H2B	109.6	C10—C11—C12	119.14 (15)
H2A—C2—H2B	108.1	C10—C11—H11A	120.4
O2—C3—C8	124.76 (14)	C12—C11—H11A	120.4
O2—C3—C4	115.40 (14)	C13—C12—C11	117.25 (16)
C8—C3—C4	119.83 (15)	C13—C12—C9	122.58 (15)
C5—C4—C3	120.37 (16)	C11—C12—C9	120.17 (14)
C5—C4—H4A	119.8	C14—C13—C12	119.13 (16)
C3—C4—H4A	119.8	C14—C13—H13A	120.4
C6—C5—C4	119.18 (15)	C12—C13—H13A	120.4
C6—C5—H5A	120.4	N3—C14—C13	124.18 (16)
C4—C5—H5A	120.4	N3—C14—H14A	117.9
C7—C6—C5	121.09 (15)	C13—C14—H14A	117.9
C7—C6—Cl1	119.78 (14)	C1—N1—N2	121.96 (14)
C5—C6—Cl1	119.11 (13)	C1—N1—H1A	117.0 (12)
C6—C7—C8	119.65 (16)	N2—N1—H1A	121.1 (12)
C6—C7—H7A	120.2	C9—N2—N1	114.93 (14)
C8—C7—H7A	120.2	C10—N3—C14	116.08 (15)
C7—C8—C3	119.88 (15)	C3—O2—C2	115.99 (12)
C7—C8—H8A	120.1		
			
O1—C1—C2—O2	−30.5 (2)	N2—C9—C12—C13	11.5 (3)
N1—C1—C2—O2	150.25 (14)	N2—C9—C12—C11	−169.00 (16)
O2—C3—C4—C5	179.56 (13)	C11—C12—C13—C14	0.6 (3)
C8—C3—C4—C5	0.6 (2)	C9—C12—C13—C14	−179.92 (17)
C3—C4—C5—C6	−0.2 (2)	C12—C13—C14—N3	−0.4 (3)
C4—C5—C6—C7	0.0 (2)	O1—C1—N1—N2	−179.81 (14)
C4—C5—C6—Cl1	−178.81 (12)	C2—C1—N1—N2	−0.5 (2)
C5—C6—C7—C8	−0.3 (2)	C12—C9—N2—N1	−179.18 (14)
Cl1—C6—C7—C8	178.51 (12)	C1—N1—N2—C9	−177.82 (15)
C6—C7—C8—C3	0.7 (2)	C11—C10—N3—C14	−0.3 (3)
O2—C3—C8—C7	−179.75 (14)	C13—C14—N3—C10	0.3 (3)
C4—C3—C8—C7	−0.9 (2)	C8—C3—O2—C2	−8.0 (2)
N3—C10—C11—C12	0.5 (3)	C4—C3—O2—C2	173.13 (13)
C10—C11—C12—C13	−0.6 (2)	C1—C2—O2—C3	−69.79 (17)
C10—C11—C12—C9	179.86 (15)		

### Hydrogen-bond geometry (Å, º)

Cg2 is the centroid of the C3–C8 phenyl ring.

**Table:**

*D*—H···*A*	*D*—H	H···*A*	*D*···*A*	*D*—H···*A*
N1—H1*A*···O1^i^	0.885 (19)	1.96 (2)	2.8423 (19)	172.9 (18)
C2—H2*A*···*Cg*2^ii^	0.97	2.88	3.676 (2)	140

Symmetry codes: (i) −*x*, −*y*+3, −*z*; (ii) −*x*+1/2, *y*+1/2, −*z*+1/2.
